# Entire Expressed Peripheral Blood Transcriptome in Pediatric Severe Malarial Anemia

**DOI:** 10.21203/rs.3.rs-3150748/v1

**Published:** 2023-07-19

**Authors:** Samuel Anyona, Qiuying Cheng, Yan Guo, Evans Raballah, Ivy Hurwitz, Clinton Onyango, Philip Seidenberg, Kristan Schneider, Christophe Lambert, Benjamin McMahon, Collins Ouma, Douglas Perkins

**Affiliations:** Maseno University School of Medicine; Center for Global Health, University of New Mexico; University of Miami; School of Public Health, Biomedical Sciences and Technology, Masinde Muliro University of Science and Technology; Center for Global Health, University of New Mexico; School of Public Health and Community Development, Maseno University; School of Medicine, University of New Mexico; University of Applied Sciences Mittweida; University of New Mexico; Los Alamos National Laboratory; Maseno University; University of New Mexico

## Abstract

This study on severe malarial anemia (SMA: Hb < 6.0 g/dL), a leading global cause of childhood morbidity and mortality, analyzed the entire expressed transcriptome in whole blood from children with non-SMA (Hb ≥ 6.0 g/dL, n = 41) and SMA (n = 25). Analyses revealed 3,420 up-regulated and 3,442 down-regulated transcripts, signifying impairments in host inflammasome activation, cell death, innate immune responses, and cellular stress responses in SMA. Immune cell profiling showed a decreased antigenic and immune priming response in children with SMA, favoring polarization toward cellular proliferation and repair. Enrichment analysis further identified altered neutrophil and autophagy-related processes, consistent with neutrophil degranulation and altered ubiquitination and proteasome degradation. Pathway analyses highlighted SMA-related alterations in cellular homeostasis, signaling, response to environmental cues, and cellular and immune stress responses. Validation with a qRT-PCR array showed strong concordance with the sequencing data. These findings identify key molecular themes in SMA pathogenesis, providing potential targets for new malaria therapies.

## INTRODUCTION

Malaria remains a significant global public health challenge with 247 million annual cases and 619,000 deaths ^1^. The majority of the cases (234 million) and mortality (593,000) occurred in the WHO African region and are due to infections with *Plasmodium falciparum*^1^, mainly in children under five of age. Kenya faces a substantial challenge with *P. falciparum* malaria, reporting ~3.42 (2.48–4.64) million annual cases and ~12,011 (10,800 – 14,000) deaths, primarily in the under-five population ^1,2^. Since the disease burden increases with transmission intensity, severe malaria remains among the leading causes of morbidity and mortality in children residing in holoendemic *P. falciparum* regions of Kenya, and other such regions of sub-Saharan Africa ^3–5^. In high transmission regions, the primary manifestation of severe malaria is severe malarial anemia [SMA, hemoglobin (Hb) < 6.0 g/dL] in both the presence and absence of respiratory distress with cerebral malaria occurring only in rare (atypical) cases ^5–7^.

The etiology of SMA is multifaceted and includes overlapping characteristics such as the destruction of infected and uninfected erythrocytes, sequestration of erythrocytes in the spleen, and suppression of bone marrow functions (reviews, see Perkins ^8,9^). Although natural immunity is acquired following repeated infections with *P. falciparum*^10–12^, innate immunity is particularly important for determining disease severity in young, malaria-naïve children. Our previous longitudinal studies in Kenyan children using a combination of candidate-gene approaches, genome-wide association studies, high-throughput genotyping, and array-based whole transcriptional profiling revealed that the development of SMA is mediated, partially by innate immune response genes ^8,13–16^. Our targeted transcriptome analyses also revealed that differentially expressed genes (DEGs) in host ubiquitination processes are a central feature of SMA pathogenesis ^17^. Microarray analysis of candidate genes in whole blood has also identified DEGs that encode amino acid transport, phospholipid metabolic processes, and positive regulation of nitrogen compound metabolic processes in Gabonese children with SMA (< 6 years old) ^18^.

Advances in high-throughput sequencing technologies and bioinformatics analyses have provided important insight into the human immune response to *P. falciparum* and identified potential vaccine candidates ^18–21^. For example, studies investigating gene expression in adults during controlled human malaria infection experiments identified > 2,700 DEGs in the whole blood transcriptome, for which a subset of 265 genes was associated with transcription and cell-cycle regulation, phosphatidylinositol signaling, and erythrocytic development ^22^. In addition, dual next-generation RNA sequencing in whole blood, which concomitantly captures host and parasite gene expression, showed that severe malaria (i.e., cerebral malaria, hyperlactatemia, or their combination) in Gambian children (< 16 years old) was associated with increased expression of granulopoiesis and interferon-γ–related genes, and suppression of type 1 interferon signaling ^20^. Despite progress in defining the human immune response to *P. falciparum*, a comprehensive investigation of the entire expressed transcriptome has not been reported in children who develop SMA, the group who suffers the highest global morbidity and mortality ^5,6,9,23–26^. Here, we present the top emergent biological processes, networks, and pathways for the first entire expressed whole blood transcriptome in Kenya children (< 5 years old) from a holoendemic region of western Kenya who develop SMA as the exclusive phenotype of severe malaria.

## RESULTS

### Demographic and Clinical Characteristics of the Study Participants

Admission demographic and clinical characteristics of the children selected for RNA-Seq in whole blood are shown in Table 1. Based on the selection criteria, sex (*p* = 0.800), overall age (*p* = 0.797), and distribution within age categories (*p* = 0.461) were comparable. Glucose levels (*p* = 0.967) and auxiliary temperature (*p* = 0.051) were comparable between the groups. Consistent with more profound anemia in children with SMA, hematocrit (*p* = 1.242E-11) and red blood cells (*p* = 1.790E-11) were lower, while red cell distribution width (*p* = 4.050E-4) and mean corpuscular volume (*p* = 0.002) were elevated. White blood cells (*p* = 1.390E-4) and lymphocyte (*p* = 1.000E-6) counts were also elevated in children with SMA. Other hematological measures were comparable between the groups, as were parasitological indices. There was a lower distribution of HbAS and higher proportion of HbSS in the SMA group (*p* = 0.029), yet not significant after multiple test correction.

### Differential Gene Expression and Central Regulatory Features in SMA

**Figure 1:**
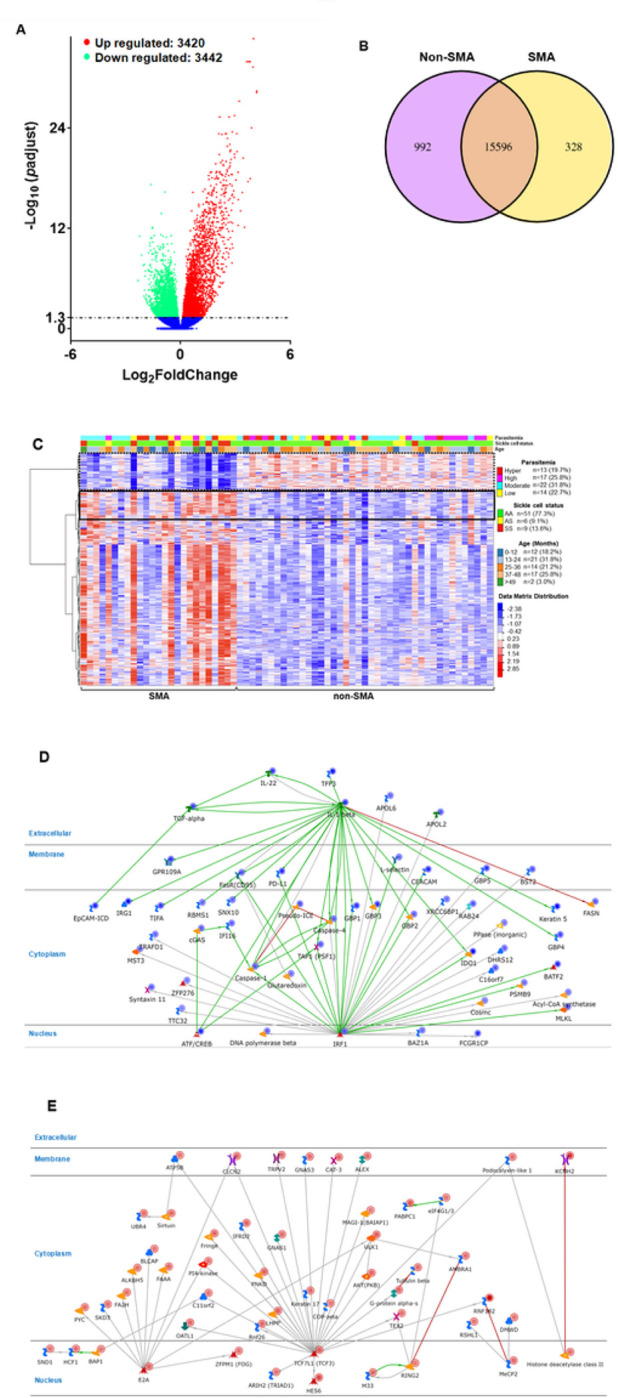
RNA-seq data for Kenyan children presenting with non-SMA (Hb≥6.0g/dL, n=41) and SMA (Hb<6.0g/dL, n=25). We used the edgeR R package (3.16.5) to infer the overall distribution of differentially expressed genes. (**A**). Volcano plot showing 3,420 up-regulated and 3,442 down-regulated protein coding genes in Kenyan children presenting with non-SMA (Hb^³^6.0 g/dL, n=41) and SMA (Hb<6.0 g/dL, n=25) cases.Horizontal axis shows the fold change of genes in different groups. Vertical axis shows the statistically significant degree of changes in gene expression levels. The points represent genes, blue dots indicate no significant difference in genes, red dots indicate up-regulated differential expression genes, green dots indicate down-regulated differential expression genes. (**B**). Venn Diagram showing Co-expression genes uniquely expressed within each clinical group, with the overlapping regions showing the number of genes that are co-expressed in two or more groups. At enrollment into the study, children with non-SMA had 992 uniquely expressed genes, while those in the SMA group had 328 genes expressed. Co-expressed genes in both clinical groups were 15,596. (**C**). Hierarchical Clustering Heatmap showing a Cluster analysis on top 1000 differential expressed genes. Hierarchical clustering analysis was carried out for log_2_(FPKM+1) of union differential expression genes in children with SMA relative to those in the non-SMA group. Genes within the same cluster show the same trends in expression levels under different clinical groups. The distribution of parasitemia, sickle cell status, and age are shown on the top for each group. The white color implies the average magnitude of gene expression. The brightest blue represents the smallest value, and the brightest red represents the highest value. Cluster 1 shown in hatched black outline and cluster 2 shown with solid black outline. (**D and E**). DEGs enrichment analysis of the process networks based on emerging clusters from the hierarchical Heatmap. The relationship between significant DEGs in the selected clusters for the SMA and non-SMA groups was determined using enrichment analysis to identify process networks on MetaCore^™^. The IRF1, IL-1β, caspase-1, caspase-4, FasR (CD95) present down-regulated genes (n=164) in cluster 1 of the heatmap (Fig. 1D), while the TCF7L1 (TCF3), E2A, RING2 network shows up-regulated (n=114) genes in cluster 2 (Fig. 1E). The blue-shaded circles show down-regulated genes and the red-shaded circles are up-regulated genes. The details of symbols used in these figures are available at: https://portal.genego.com/legends/MetaCoreQuickReferenceGuide.pdf.

Differential expression analysis identified 3,420 up- and 3,442 down-regulated genes (*p*adj < 0.050) in children with SMA (Fig. 1A). Comparison of the DEGs revealed 992 genes that were uniquely expressed in non-SMA, 328 in SMA, and 15,592 co-expressed genes (Fig. 1B). A non-supervised hierarchical cluster analysis of the top 1000 DEGs identified unique co-regulated gene clusters in children with SMA, and computed the distribution of clinical variables (i.e., parasitemia, sickle cell status, and age) in each of the groups (Fig. 1C).

Canonical pathway maps for direct functional interactions were generated to gain insight into the networks for two of the major clusters, one down-regulated (n = 156, cluster 1) and one up-regulated (n = 114, cluster 2). The network for the down-regulated genes (cluster 1) was IRF1↔IL-1β↔Caspase-1↔Caspase-4↔FasR with the transcription factor, IRF1, as the central divergence hub (36 direct interactions) and IL-1β as the central convergence hub (27 direct interactions, Fig. 1D). The top process associated with the functional interactions for cluster 1 was Response to Stress (*p* = 2.404E-08). Cluster 2 (up-regulated) generated a TCF7L1↔E2A↔RING2 network with two transcription factors, TCF7L1, as the central divergence hub (26 direct interactions) and E2A, as a secondary divergence hub (10 functional interactions). The central convergence hub in cluster 2 was RING2 with 5 functional interactions (Fig. 1E). Regulation of Metabolic Process was the top represented process for cluster 2 (*p* = 2.671E-09). Collectively, these results suggest that SMA is characterized by enhanced stress responses and perturbations in metabolic processes.

### Altered Leukocytic Immune Cell Profiles in SMA

**Figure 2:**
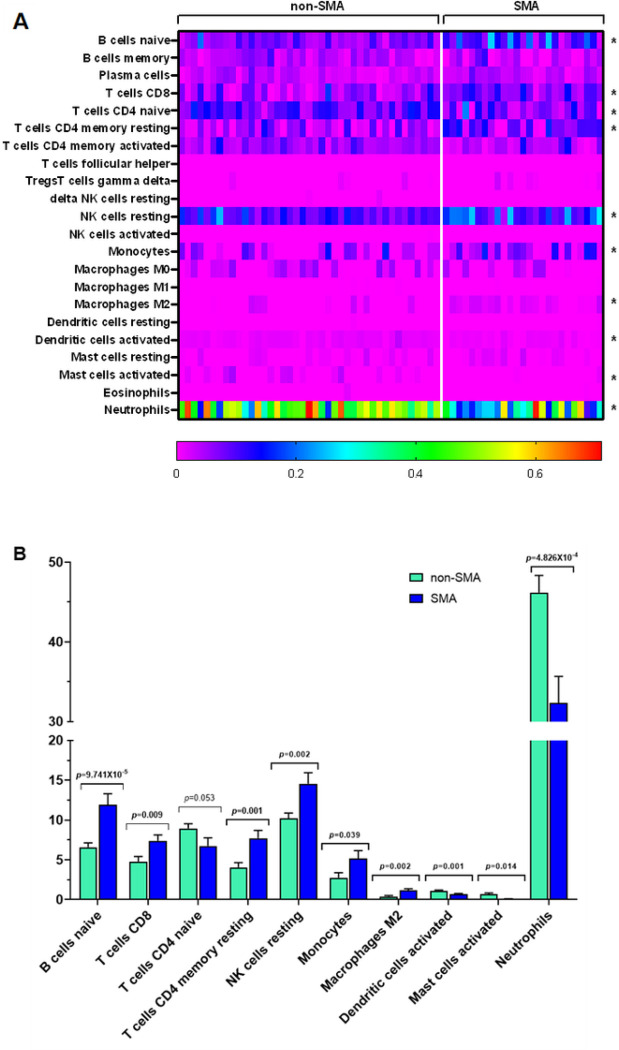
Estimation of Immune Cell Type Proportions in Whole Blood. Deconvolution analysis of the different cell types in blood was determined using CIBERSORTx. Cellular frequencies were imputed using LM22 as the signature matrix file. (**A**) Heatmap representing the cell type expression for 22 types/subtypes of leukocyte cell populations presented at the individual patient level in the non-SMA (Hb^³^6.0 g/dL, n=41) and SMA (Hb<6.0 g/dL, n=25) groups. *Indicates significant differences (*p*<0.050) in immune cell proportions between the two groups determined using two-sided, two-sample t-tests with Welch correction. (**B**) Relative proportion (%) of expression for the immune cell types that differed significantly between children with non-SMA and SMA. Bivariate analysis was performed using two-sided, two-sample t-tests with Welch correction and presented as mean (SEM) for the non-SMA and SMA groups.

To determine if leukocytic immune profiles differed in children who developed SMA, a bioinformatic approach was implemented using CIBERSORTx. Although there was interindividual variability, 10 immune cell types were differentially expressed at *p* < 0.050 (Fig. 2A). Children with SMA had increased expression of naïve B cells (*p* = 9.741E-05), CD8 T cells (*p* = 0.009), CD4 memory resting T cells (*p* = 0.001), resting NK cells (*p* = 0.002), monocytes (*p* = 0.039), and M2 macrophages (*p* = 0.002) (Fig. 2B). In contrast, the SMA group had a lower proportion of expression for activated dendritic cells (*p* = 0.001), activated mast cells (*p* = 0.014), and neutrophils (*p* = 4.826E-04), along with marginally reduced expression of naïve CD4 T cells (*p* = 0.053) (Fig. 2B). The immune cell type patterns observed indicate that children with SMA have a decreased antigenic response, reduced immune priming, and enhanced polarization towards cellular proliferation and tissue repair.

### Functional Enrichment Analysis Reveals Distinct Features of SMA

**Figure 3:**
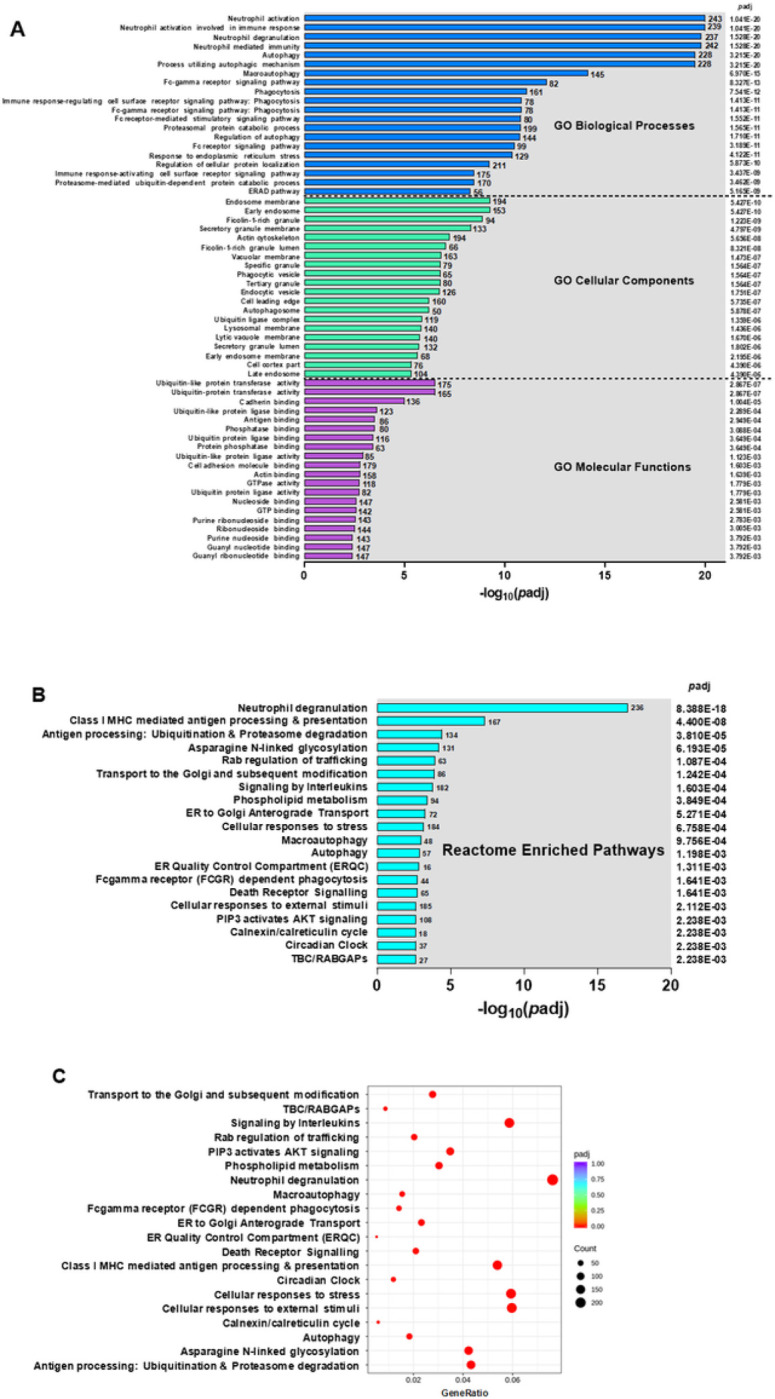
Functional enrichment analysis. (A) GO Enrichment Analysis showing the top 20 enriched terms in the biological process, cellular component, and molecular function categories of DEGs in children with SMA (Hb<6.0 g/dL, n=25), relative to those in the non-SMA (Hb≥6.0 g/dL, n=41) group. GO enrichment analysis was done using the clusterProfiler R package, while correcting for bias on gene length. GO terms of enriched DEGs with *p*-adjusted values <0.050 were considered significantly. The X-axis represents the negative log_10_ of *p*-adjusted (−log_10_[*p*-adjusted) values. (B) Reactome enrichment analysis of top 20 enriched terms that were significantly different in children with SMA, relative to those with non-SMA. (C) Reactome enrichment histogram of the top 20 terms. The Y-axis indicates the pathway name. The X-axis represents the gene ratio of up- and down-regulated genes. The size of the black dots corresponds to the number of genes annotated, and the depth of the red color implies magnitude of enrichment (*p*-adjust)

To identify biological processes characteristic of developing SMA, enrichment analysis was performed. Gene ontology (GO) enrichment analysis was performed for three domains (i.e., biological process, cellular components, and molecular functions) with the top 20 in each presented in Fig. 3A. Of the three domains, biological processes showed the greatest enrichment in children with SMA, for which altered neutrophil responses (*p*adj = 1.041E-20 to 1.528E-20) and autophagy-related processes (*p*adj = 3.215E-20 to 6.970E-15) ranked among the highest. The top cellular components enriched in SMA included endosome responses (*p*adj = 5.427E-10 to 5.427E-10). For molecular functions, ubiquitin-related process showed the greatest enrichment (*p*adj = 2.867E-07 to 1.779E-03). Consistent with GO process results, the top-ranked Reactome enriched pathways in children with SMA were neutrophil degranulation (*p*adj = 8.388E-18), class I MHC-mediated antigen processing and presentation (*p*adj = 4.400E-08), and antigen processing involving the ubiquitination and proteasome degradation (*p*adj = 3.810E-05, Fig. 3B and C). Results from the GO and Reactome enrichment analyses converge on common biological processes and suggest that SMA is characterized by altered neutrophil responses, perturbations in autophagy and endosomal pathways, and activation of ubiquitin-related processes.

#### KEGG Canonical Pathways Implicated in SMA:

**Figure 4:**
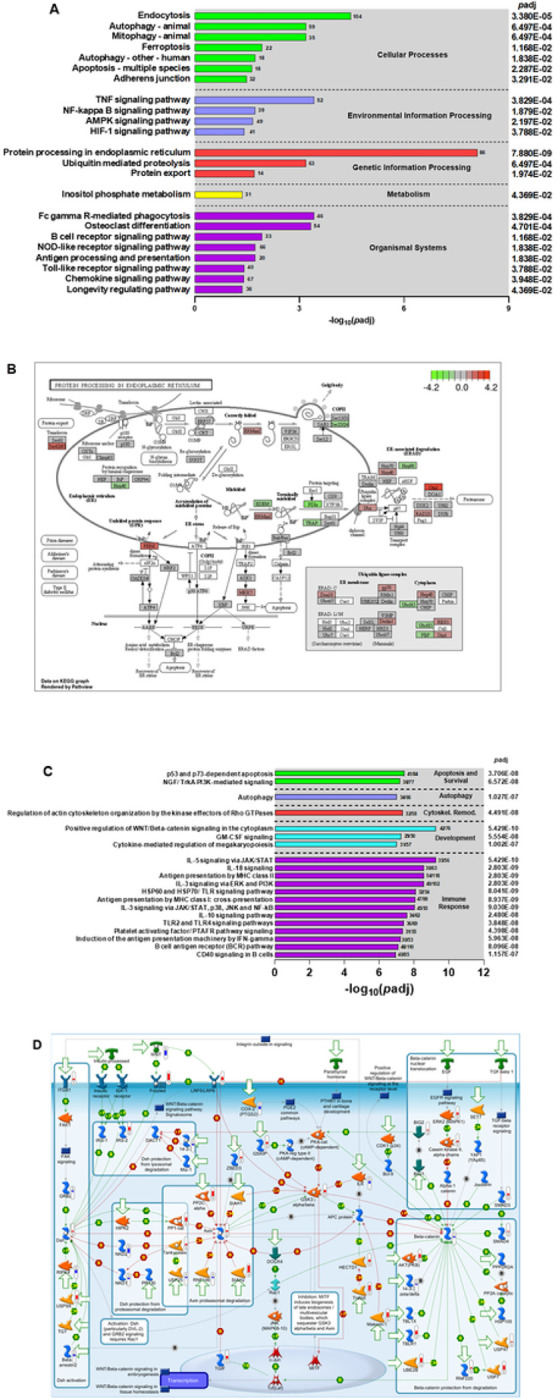
Canonical pathway analysis of DEGs. (**A**) Functional classification of KEGG pathway of the DEGs between non-SMA (Hb^³^6.0 g/dL, n=41) and SMA (Hb<6.0 g/dL, n=25) groups. The KEGG terms were grouped into 5 categories, namely; (i) cellular processes, (ii) environmental information processing, (iii) gene information processing, (iv) metabolism, and (v) organismal systems. The left Y-axis shows the KEGG terms. The right Y-axis shows *p*-adjusted values for each KEGG term. The X-axis represents the negative log 10 of *p*-adjusted values (−log_10_[*p*-adjusted]). (**B**) The top emerging KEGG term was the protein processing in the endoplasmic reticulum pathway. DEGs mapped to the pathway in children with SMA relative to non-SMA groups. Red boxes show genes that were up-regulated in children with SMA and green boxes were genes down-regulated in SMA cases relative to controls. (**C**) Distribution of top 20 gene ontology (GO) terms using Metacore^™^. The GO terms were classified into 5 categories; (i) apoptosis and survival, (ii) autophagy, (iii), cytoskeleton remodeling, (iv) development and (v) immune response. The left Y-axis shows the GO terms. The right Y-axis shows *p*-adjusted values for each GO term. The X-axis represents the −log_10_[*p*-adjusted. (**D**) A schematic model of the top GO enrichment term, the positive regulation of WNT/Beta-catenin signaling in the cytoplasm. The pathway map was generated using MetaCore^™^. The red color thermometers show annotated genes that were up-regulated in children with SMA. Blue colored thermometers show genes that were down-regulated in cases versus controls. The details of symbols used in these figures are: https://portal.genego.com/legends/MetaCoreQuickReferenceGuide.pdf.

Functional classification of the DEGs between SMA and non-SMA for the KEGG pathways is shown in Fig. 4A. The top-ranked pathways were: (i) cellular processes - endocytosis (*p*adj = 3.380E-05); (ii) environmental information processing - TNF signaling (*p*adj = 3.829E-04); (iii) genetic information processing - protein processing in ER (*p*adj = 7.880E-09), which was also the most significant of all pathways; (iv) metabolism - inositol phosphate metabolism (*p*adj = 4.369E-02); and (v) organismal systems - FcγR-mediated phagocytosis (*p*adj = 3.829E-04). Collectively, the KEGG results suggest that children with SMA exhibit significant alterations in immune response triggering, cellular recycling processes, and protein regulation.

#### Endoplasmic Reticulum Quality Control Dysfunction in SMA:

To further characterize the pathogenesis of SMA, we focused on the top emergent pathway generated from the KEGG database: Protein Processing in Endoplasmic Reticulum (ER; Fig. 4B). Children with SMA had elevated expression of ERManI and PERK and decreased expression of HSP40, EDEM, PDIs, and TRAP, indicating that the ER quality control system is impaired, leading to potential protein misfolding and cellular dysfunction. This is consistent with up-regulation of several transcripts in the ubiquitin ligase complex in the ER membrane (i.e., Ubx, gp78, and Derlin1) and dysregulation in the ER cytoplasm (i.e., elevated HSP40, RBX1, and Skp1, and decreased UbcH5 and FBP). Because of misfolded or damaged proteins, the ER-associated degradation pathway was activated, as illustrated by increased Otu1 and RAD23 expression. Further indications of ER-associated cellular stress and attempts to maintain cellular homeostasis in children with SMA are demonstrated by elevated Sec62/Sec63 complex subunits and MKK7.

#### MetaCore^™^ Canonical Pathways Implicated in SMA:

Additional characterization of SMA pathogenesis was carried out by exploring the top 20 pathway maps generated with MetaCore^™^ (Fig. 4C). The top-ranked pathways were: (i) apoptosis and survival - p53/p73-dependent apoptosis (*p*adj = 3.706E-08); (ii) autophagy – autophagy (*p*adj = 1.027E-07); (iii) cytoskeleton remodeling - regulation of actin cytoskeleton organization by the kinase effectors of Rho GTPases (*p*adj = 4.491E-08); (iv) development - positive regulation of WNT β-catenin signaling (*p*adj = 5.429E-10); and (v) immune response - IL-5 signaling via JAK/STAT (*p*adj = 5.429E-10). These results show that the pathogenesis of SMA is a complex and multi-faceted process that involves multiple molecular mechanisms involved in cell growth and differentiation, as well as cellular and immune stress responses.

### Deciphering Cellular Stress Responses in SMA

Of the top canonical pathways that emerged from MetaCore^™^, we focused on positive regulation of WNT/β-catenin signaling to further define cellular and immune stress responses in SMA (Fig. 4D). Children with SMA had reduced expression of WNT, but increased expression of the WNT receptor, LRP5/LRP6, and its target, Axin, which was also signaled by increased expression of ZBED3, SIAH2, PP2C, PP1-cat, and tankyrases. This transcriptional pattern indicates enhanced proteasomal degradation in the context of reduced protection from degradation (i.e., down-regulation of NKD1 and NKD2), despite increased expression of Dsh signaled by up-regulation of the WNT receptor (i.e., Frizzled), transmembrane receptor (i.e., ITGB1), IRS-2, and USP9X, accentuated by decreased phosphorylation from RIPK4 and reduced binding by GRB2 and β-aresstin2. Children with SMA also had increased expression for SMAD3, SMAD4, TBLR1, Makorin-1, Trabid, UBE2B, RNF220, USP7, PKB, and USB47 in the context of decreased expression in β-catenin, indicating activation of the non-canonical WNT signaling pathway. This pattern of expression also suggests cellular stress responses and attempts to regulate protein degradation and stabilize cell survival through alterations in ubiquitination and de-ubiquitination processes.

#### Validation of Whole Blood Transcriptome Data using a Targeted Gene Array:

**Figure 5:**
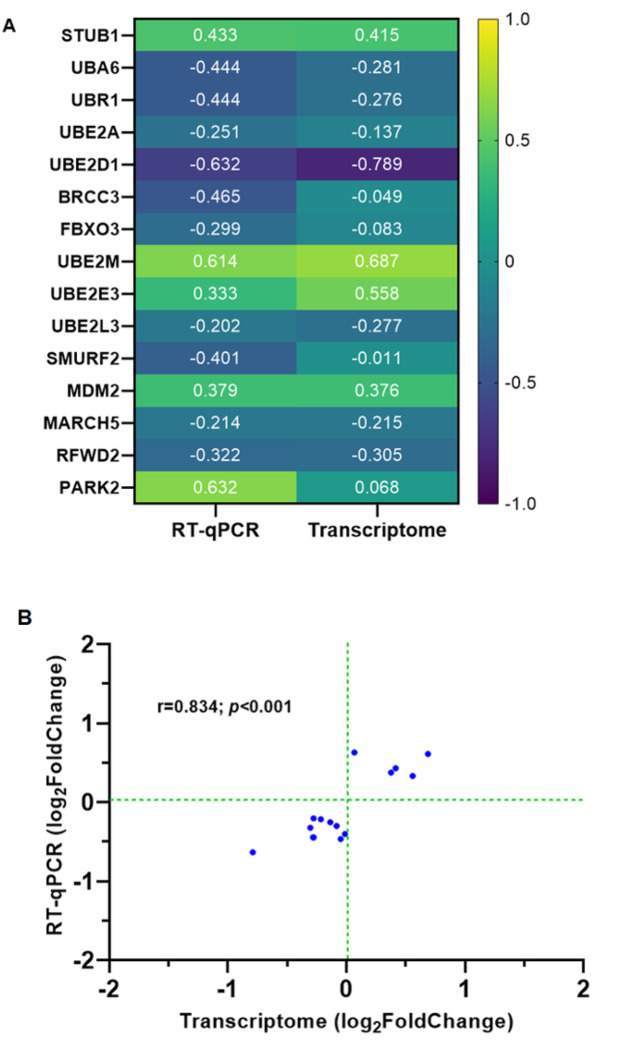
Validation of the transcriptome data. A Human Ubiquitination Pathway RT^2^ Profiler PCR Array kit (Qiagen, LLC-USA, Germantown, MD, United States) was used to measure the expression of genes involved in the ubiquitination process (A) Heat map shows a graphical and color-coded representation of fold regulation (Log_2_) comparison of significant DEGs using the RT-qPCR array versus data generated from the whole blood transcriptome analysis. The Y-axis shows the gene names. The X-axis shows the assay type. The darkest purple represents the lowest fold change, and the brightest yellow represents the highest fold change in children with SMA relative to non-SMA. (B) Correlation scatter plot of the significantly expressed ubiquitination process genes in the RT-qPCR analysis (Y-axis) versus the transcriptome data (X-axis). There was a strong positive correlation of the DEGs in SMA cases (r=0.834, *p*<0.001).

To validate the RNA-Seq results, we utilized a targeted gene approach with a qRT-PCR array that captured 84 genes involved in ubiquitination. A heatmap cluster analysis showed strong concordance in the fold-change and directionality in the two platforms (Fig. 5A). Cluster analysis of the significant DEGs in the qRT-PCR array showed a significant correlation with the same genes captured from RNA-Seq (r = 0.834, *p* < 0.001; Fig. 5B). To further compare findings from the two datasets, a workflow was generated in MetaCore^™^ to identify common (shared) genes that mapped to pathways, GO processes, and process networks. The combined data objects (gene set) from the two platforms revealed the following top-ranked features: (i) pathway map – Proteolysis Ubiquitination (*p*adj = 2.193E-11); (ii) GO process – Protein ubiquitination (*p*adj = 1.083E-12); and (iii) process network - Proteolysis Ubiquitin - proteasomal proteolysis *p*adj = 2.250E-05) (**Supplemental Fig. 1A-C**).

## DISCUSSION

Severe life-threatening malaria is represented by distinct and overlapping disease features (one or more) of the following: impaired consciousness, prostration, multiple convulsions, acidosis, hypoglycemia, SMA, renal impairment, jaundice, pulmonary edema, significant bleeding, shock, and hyperparasitemia ^27–33^. Identifying common gene pathways/networks that encompass the diverse pathophysiological landscape of severe malaria (i.e., mixed phenotype) has presented significant challenges, likely because distinct biological processes may not share common networks. The clinical manifestations of severe malaria and the age at which they present are largely driven by *P. falciparum* endemicity ^9,34^. The overwhelming majority of life-threatening severe malaria occurs in holoendemic *P. falciparum* transmission areas of sub-Saharan Africa in children under five years who develop SMA, making this severe manifestation a leading cause of childhood deaths in such regions ^8,9^.

A major advantage of studies in holoendemic malaria regions, such as western Kenya, is that children have a distinct pathophysiological presentation of SMA, making the discovery of gene-disease relationships more feasible. Our previous studies have identified innate immune response genes that influence the pathogenesis of SMA, largely through imparting changes in soluble mediators of inflammation ^8,9,15,35,36^. However, this is the first investigation to examine the entire expressed peripheral blood transcriptome in children whose primary phenotype of severe disease is SMA. Findings from this study identify novel biological pathways and process networks that converge on perturbations in cellular and immune stress responses, illustrating that the pathogenesis of SMA is complex and multi-faceted.

Molecular patterns in children with SMA were initially identified by hierarchical clustering of the top 1000 DEGs. Relationships that emerged from the analysis were then further deciphered by creating canonical process networks for several gene clusters. Cluster 1 (IRF1↔IL-1β↔Caspase-1↔Caspase-4↔FasR) contained a set of down-regulated genes that are central to host inflammasome activation, cell death, and innate immune responses ^37^. Children with SMA had a decrease in the transcription factor, IRF1, and downstream targets (i.e., IL-1β, capsases 1 and 4, and FasR) in the absence of a Nod-like receptor family (i.e., NLRP1, NLRP3, and NLRC4) response ^37–39^. This suggests an inability to initiate pyroptosis and dysregulated inflammasome. Cluster 2 (TCF7L1↔E2A↔RING2) was up-regulated in SMA and contained a family of genes involved in cellular stress responses ^40,41^. This set of genes also emerged in the top-ranked pathway (i.e., positive regulation of WNT/β-catenin signaling) suggesting that SMA is characterized by increased cellular stress responses via enhanced proteasomal degradation in the context of reduced protein degradation through the non-canonical WNT signaling pathway.

Inheritance of sickle cell trait (HbAS) has protective effects against the development of severe malaria ^42–46^. As expected in a holoendemic region, there was a higher proportion of HbSS carriage (sickle cell disease) in the SMA group. Since hierarchical clustering analysis indicated more pronounced gene dysregulation in children with HbSS, the analyses were repeated without these children. This resulted in an identical network of down-regulated genes (cluster 1), but a different set of up-regulated genes (TAL1↔LYL1↔EKLF1↔HMBS↔RHD) that are involved in the regulation and maturation of erythrocytes and Hb production (review, see Love ^47^). Thus, there are both overlapping and distinct molecular profiles in children who develop SMA with and without inheritance of HbSS (**Supplemental Fig. 2A-C**).

Results from the immune profiling with CIBERSORTx indicate that children with SMA have a decreased antigenic response, reduced immune priming, and an enhanced polarization towards cellular proliferation and repair. The hematological patterns captured by the CBC, although not as specific, parallel results obtained from the immune cell profiling. Consistency between the two independent methods supports the reliability of the observed immune alterations in SMA.

To gain further insight into SMA pathogenesis, we used a combination of functional enrichment analysis platforms to identify convergent patterns amongst central themes. One distinct feature of SMA was neutrophil activation and degranulation. Although not specifically in children with SMA, previous studies suggest that neutrophil activation is more pronounced in severe malaria ^20,48–53^. An additional molecular convergence among children with SMA was perturbations in autophagy. Altered autophagy, to our knowledge, has not been described in human malaria pathogenesis, but autophagy defects appear common in various other infectious diseases (review, see Deretic ^54^).

The transcriptome in children with SMA also revealed dysregulation in proteasome-mediated activity. This finding parallels our earlier studies that were the first to report DEG in ubiquitin-related processes as a feature of SMA ^17,55^. Canonical pathway analyses showed additional molecular features of SMA including changes in cellular homeostasis, signaling, environmental response, and various molecular mechanisms that regulate cellular and immune stress responses. A key emergent pathway in SMA was protein processing in the ER with DEGs involved in protein folding, export, targeting, ERAD, and the ubiquitin ligase complex. While these pathways have not been described for the human response to malaria, similar pathways are operational in human malaria parasites and are being explored as novel therapeutic targets ^56–58^.

Changes in endocytosis-mediated pathways were also witnessed in SMA, indicating significant changes to cellular structure as a molecular theme, as has been described in experimental murine cerebral malaria models ^59^. The central role of cellular stress response disruptions in SMA is further supported by dysregulation in the WNT/β-catenin signaling pathway. While changes in this pathway have been implicated in various diseases and are known to be affected by antimalarial drugs ^60–64^, its role in immune response to malaria is a novel finding. Validation of the transcriptome data was performed using a targeted human ubiquitination qRT-PCR array technology containing 84 genes. We compared 15 significant DEGs previously identified in children with SMA ^17^, and observed a high level of concordance and identical directionality from the two platforms. Moreover, enrichment analyses using the full datasets for both platforms revealed highly consistent and shared process networks and pathways’, providing further validation and confirmation that disruptions in the ubiquitin proteasome system is a fundamental feature of SMA pathogenesis.

In conclusion, an unbiased RNA-Seq analysis capturing the entire expressed blood transcriptome identified key molecular aspects of SMA pathogenesis, such as changes in neutrophil responses, autophagy, endosomal pathways, and activation of ubiquitin-related processes and cellular stress responses. Strengths of this study are the extensive clinical characterization of the cohort which allowed for the exclusion of co-infections known to influence the immune response ^65–67^, and a robust sample size. Limitations of the study include potential generalizability to other forms of severe malaria, such as cerebral malaria, which is likely a distinct pathogenesis. To determine the impact of the observed transcriptional changes on protein expression, we are currently performing proteomics in the cohort presented here. Collectively, results presented here highlight the complexity and multi-dimensional nature of SMA. An improved understanding of this complexity can guide the development of novel targeted therapies for improved clinical outcomes.

## METHODS

### Study region and participants

This study was conducted at Siaya County Referral Hospital (SCRH), located in a holoendemic *P. falciparum* transmission region in western Kenya where SMA is among the main causes of childhood morbidity and mortality in the community ^5,6,25,26,68–70^. Individuals inhabiting the study area are predominantly from the Luo group (> 96%), an ethnically homogeneous population ^71^. Children presenting at SCRH with symptoms of infectious diseases, and who met the following inclusion criteria; auxiliary temperature ≥ 37.5°C (axillary), age 0–59 mos., distance to hospital ≤ 25 km, and parent/guardian willing and able to provide signed informed consent and attend follow-up visits (14 days later), were approached for enrollment into the study. Children presenting with suspected non-infectious diseases were excluded. Based on inclusion/exclusion criteria, 565 children were enrolled into the acute febrile cohort (3/2017 to 9/2020). For children requiring hospitalization and those released as outpatients, venipuncture blood samples (3–4mL) were collected on admission into the study (day 0), prior to treatment with antimalarials or other medication. The study was approved by the Kenya Medical Research Institute Scientific and Ethics Review Unit, the University of New Mexico Institutional Review Board, and the Maseno University Scientific and Ethics Review Committee. Written informed consent was provided by the parents/legal guardian of the study participants.

### Laboratory procedures

At enrollment, demographic and clinical data were collected, and a physical examination performed. Giemsa-stained thin blood smears were prepared, examined, and asexual malaria parasite densities determined ^25^. Complete blood counts (CBCs) were determined using a DxH 500 hematology analyzer (Beckman-Coulter). Since we have shown that co-infections influence malarial anemia severity in the Siaya community, all children were tested for HIV-1 and blood-borne bacterial infections according to our previously described methods ^65–67^. Parents/legal guardians of participating children received pre- and post-test HIV&AIDS counseling. To further characterize potential causes of anemia, sickle-cell trait status was determined by alkaline cellulose acetate electrophoresis (Helena BioSciences).

### Study participant selection for RNA-Seq

For selection of samples for the RNA-Seq from the 565 enrolled study participants, children with malaria were stratified into two groups based on Hb levels (i.e., Hb ≥ 6.0 g/dL and Hb < 6.0 g/dL), and then matched according to age and sex. Further selection criteria for the RNA-Seq included omitting children with any detected co-infections (e.g., HIV, tuberculosis, bacteremia, etc) ^65–67^. This selection strategy yielded 41 children with non-SMA (Hb ≥ 6.0 g/dL) and 25 children with SMA (Hb < 6.0 g/dL).

### RNA isolation, library construction, and sequencing

Approximately 500μL of whole blood collected from venipuncture prior to treatment was stabilized with Trizol^®^ (Thermo Fisher Scientific Inc.), immediately frozen in liquid nitrogen, and then subsequently stored at −80°C. Total RNA was batch-isolated using E.Z.N.A^®^ Total RNA Kit (Omega Bio-Tek Inc.), treated with RNase-free DNase I (New England Biolabs Inc.), and further processed using RNA Clean & Concentrator (ZYMO Research Corp.). Prior to library preparation and sequencing, RNA degradation and contamination were captured on agarose gels with purity confirmed using a NanoPhotometer^®^ (IMPLEN). RNA integrity and quantification were measured using the RNA Nano 6000 Assay Kit on a Bioanalyzer 2100 system (Agilent Technologies). To capture the entire expressed transcriptome, sequencing libraries were generated using NEBNext^®^ Ultra^™^ RNA Library Prep Kit for Illumina (NEB) following the manufacturer’s protocol. Clustering of index-coded samples was performed on a cBot Cluster Generation System using PE Cluster Kit cBot-HS (Illumina). Sequencing was performed to a depth of > 20 million high-quality mappable reads on an Illumina NovaSeq 6000 sequencer (Novogene Corporation Inc.). Raw reads of FASTQ format were processed to obtain clean reads used in the downstream analyses.

### Bioinformatics analysis

Raw data were quality-controlled and filtered using fastp ^72^ and aligned to the human reference genome (GRCh38.p13) ^73^ using STAR version 2.5 ^74^. HTSeq v0.6.1 ^75^ was used to generate read counts for individual transcripts for each sample with mRNA abundance normalized as Fragments Per Kilobase Million Reads (FPKM) of each gene ^19^. *P*-values were adjusted using the Benjamini Hochberg procedure ^76^.

Differential expression analysis: Differential expression analysis of the two clinical conditions was performed using edgeR (3.16.5). For this study, adjusted *p*-values (*p*adj) of < 0.050 plus absolute log_2_-(fold-change) of > 1 were used as the threshold for DEGs. To identify the correlation between different genes, samples were clustered using expression level FPKM utilizing the hierarchical clustering distance method.

Leukocytic immune cell profiling: The relative percentage of different immune cell types/subtypes in whole blood was imputed using CIBERSORTx ^77,78^. This analytical tool processes gene expression data from a bulk admixture of different cell types to estimate the abundance of member cell types in a mixed cell population ^79^. The curated signature matrix file, LM22, was used as the reference to deconvolute the relative fraction of different cell types in whole blood, resulting in inference of 22 types/subtypes of leukocytes. Imputation of cell-type specific gene expression levels were performed at the sample-level with the output presented as the fractional proportion in whole blood for each study participant. The relative proportions of immune cell types were then compared between the non-SMA and SMA groups.

Enrichment analysis: ClusterProfiler ^80,81^ R package was used to implement the enrichment analysis. Gene Ontology (GO) analysis of DEGs was used to infer functional and biological functions, correcting for gene length bias ^81^. Reactome Enrichment Analysis was used to identify pathways that mapped to biological and cellular networks ^82^. Significantly enriched pathways were identified with Kyoto Encyclopedia of Genes and Genomes (KEGG) using the R package ‘ClusterProfile’ ^81^. For all analysis, *p*adj < 0.050 were considered significant enrichment. Confirmation and further discovery of the findings were implemented by using MetaCore^™^ (https://clarivate.com/products/metacore/) to identify DEGs that mapped to GO processes, process networks, and pathways.

### Validation of transcriptome profiles

To validate the transcriptome data, we used data generated from a targeted gene approach that measured transcript expression levels of 84 key genes involved in the ubiquitination process (Human Ubiquitination Pathway RT^2^ Profiler PCR Array kit, Qiagen). Total RNA was isolated from whole blood samples from children with non-SMA (Hb ≥ 6.0 g/dL, n = 23) and SMA (Hb < 6.0 g/dL, n = 21) who were not included in the RNA-Seq. Cluster analysis was used to compare the significantly expressed genes in the quantitative reverse transcription polymerase chain reaction (qRT-PCR) array with identical genes generated from the RNA-Seq. Convergence between the two datasets was determined by the ‘compare experiments workflow’ in MetaCore^™^ for GO processes, process networks, and pathways.

## Figures and Tables

**Table 1:** Demographic and clinical characteristics of the study participants.

Characteristics	Total	non-SMA (Hb ≥ 6.0 g/dL)	SMA (Hb < 6.0 g/dL)	*p*-value
No. of participants, n	66	41	25	
Sex, n (%)				
Male	33 (50.0)	20 (48.8)	13 (52.0)	0.800^a^
Female	33 (50.0)	21 (51.2)	12 (48.0)	
Age, months	24.5 (23.3)	24.0 (22.0)	25.0 (28.5)	0.797^b^
0–12.9	12 (18.2)	7 (17.1)	5 (20.0)	0.461
13–24.9	21 (31.8)	14 (34.1)	7 (28.0)	
25–35.9	14 (21.2)	9 (22.0)	5 (20.0)	
36–48.9	17 (25.8)	11 (26.8)	6 (24.0)	
>49	2 (0.0)	0 (0.0)	2 (8.0)	
Glucose, mmol/L	5.0 (2.0)	5.0 (2.3)	5.0 (1.7)	0.967^b^
Admission temperature, °C	37.9 (1.1)	38.0 (1.2)	37.7 (0.8)	0.051^b^
**Hematological Parameters**				
Hemoglobin, g/dL	9.2 (5.2)	9.9 (1.4)	4.6 (1.2)	NA
Hematocrit, %	25.1 (15.8)	29.8 (5.9)	14.4 (2.9)	**1.242E-11** ^b^
Red blood cells, × 10^6^/μL	3.5 (2.5)	4.3 (1.0)	1.9 (0.9)	**1.790E-11** ^b^
Red cell distribution width, %	19.8 (5.9)	18.7 (3.4)	22.3 (8.9)	**4.050E-4** ^b^
Mean corpuscular volume, fL	71.0 (14.2)	69.5 (9.2)	78.6 (29.9)	**0.002** ^b^
Mean corpuscular hemoglobin, pg	23.9 (6.3)	22.9 (4.8)	26.7 (9.4)	0.022^b^
Mean corpuscular hemoglobin concentration, g/dL	31.9 (5.9)	32.4 (6.7)	31.4 (5.6)	0.372^b^
Platelets, ×10^3^/μL	130.3 (99.2)	124.4 (85.7)	134.0 (139.7)	0.615^b^
Platelet distribution width, %	16.9 (1.2)	16.5 (1.3)	17.3 (0.9)	0.730^c^
Mean platelet volume, fL	8.6 (1.8)	8.5 (1.6)	8.9 (1.9)	0.124^b^
WBCs, ×10^3^/μL	12.7 (10.3)	11.3 (6.9)	19.8 (11.5)	**1.390E-4** ^b^
Lymphocytes, ×10^3^/μL	4.2 (4.7)	3.7 (1.6)	10.0 (9.2)	**1.000E-6** ^b^
Monocytes, ×10^3^/μL	1.4 (1.3)	1.2 (1.3)	1.7 (1.4)	0.022^b^
Neutrophils, ×10^3^/μL	5.4 (6.1)	5.3 (4.2)	6.0 (6.9)	0.438^c^
Granulocytes, ×10^3^/μL	7.0 (4.3)	6.7 (3.0)	9.1 (5.8)	0.373^c^
**Parasitological Indices**				
Parasite density, MPS/μL	38250 (82263)	57915 (81568)	14191 (68728)	0.155^b^
Low (1–5,000)	14 (21.2)	6 (14.6)	8 (32.0)	0.134^a^
Moderate (5001–50,000)	23 (34.8)	13 (31.7)	10 (40.0)	
High (50,001–100,000)	17 (25.8)	14 (34.1)	3 (12.0)	
Hyper (> 100,001)	12 (18.2)	8 (19.5)	4 (16.0)	
Geomean parasitemia, /μL	23,647	28,888	17,029	0.447^c^
**Genetic Variants**				
Sickle cell trait, n (%)				0.029^a^
Hb AA	51 (77.3)	35 (85.4)	16 (64.0)	
Hb AS	6 (9.1)	4 (9.8)	2 (8.0)	
Hb SS	9 (13.6)	2 (4.9)	7 (28.0)	

Data are the median (interquartile range; IQR) unless otherwise noted. Children (n = 66) presenting with malaria at SCRH were recruited. Based on hemoglobin (Hb) levels, children were categorized into either non-severe malaria anemia (non-SMA; Hb ≥ 6.0 g/dL, n = 41) or severe malarial anemia (SMA; Hb < 6.0 g/dL, n = 25).

a Fisher’s exact test with exact *p*-values for homogeneity was performed.

b Two-sided Mann-Whitney-U tests were used to compare the non-SMA and SMA groups

c Group means were compared by two-sided, two-sample t-test, with equal variance.

All *p*-values shown in bold remained below the significance level after multiple test correction using the Bonferroni-Holm method (familywise error rate, significance level 0.050).

Abbreviations: MPS - malaria parasites presented as mean (standard deviation).

## Data Availability

The datasets generated and analyzed during the current study are available on reasonable request. For original data please contact dperkins@salud.unm.edu.
